# Effects of temperature on the development and population growth of the melon thrips, *Thrips palmi* , on eggplant, *Solanum melongena*

**DOI:** 10.1093/jis/14.1.78

**Published:** 2014-01-01

**Authors:** Ramchandra Yadav, Niann-Tai Chang

**Affiliations:** 1 Department of Tropical Agriculture and International Cooperation, National Pingtung University of Science and Technology, Pingtung, Taiwan R.O.C. 91201; 2 Department of Plant Medicine, National Pingtung University of Science and Technology, Pingtung, Taiwan R. O. C. 91201

**Keywords:** fecundity, life table, thermal constant, threshold temperature

## Abstract

The effects of temperature on the melon thrips, *Thrips palmi* Karny (Thysanoptera: Thripidae), preimaginal development, survival, fecundity, longevity of females and males, and population growth were investigated at 16, 19, 22, 25, 28, and 31° C, 70–80% RH, and a photoperiod of 12:12 L:D. The results indicated that the duration of egg, larval, and pupal stages was significantly influenced by increased temperature. The egg-to-adult developmental period of *T. palmi* declined from 35.7 to 9.6 days as the temperature increased from 16 to 31° C. The developmental threshold temperature estimated for egg-to-adult was 11.25° C, with a thermal constant of 196.1 degreedays. The developmental threshold temperature was 13.91, 11.82, 9.36, and 10.45° C for adult preoviposition period, total preoviposition period, female longevity, and male longevity, respectively. The thermal constants for completing the adult preoviposition period, total preoviposition period, female longevity, and male longevity were 29.3, 227.3, 454.6, and 344.8 degreedays, respectively. Female longevity was found to be shortest at 31° C (18.7 days) and longest at 16° C (56.7 days), and male longevity was shortest at 31° C (15.5 days) and longest at 16° C (50.7 days). Fecundity was highest at 25° C (64.2 eggs/female) and lowest at 16° C (23.4 eggs/female). The population trend index of *T. palmi* was highest at 25° C (31.3) and lowest at 16° C (7.6). The optimal developmental temperature for *T. palmi* in eggplant, *Solanum melongena* L. (Solanales: Solanaceae), was determined to be 25° C.

## Introduction

Temperature is one of the most critical environmental factors influencing rate of insect growth and development ( Taylor 1981 ). Developmental rate is usually used to quantify the effect of temperature. Previous studies have shown that temperature regulates seasonal and daily cycles, and thus indirectly influences various aspects of insect biology, such as sex ratio ( Zheng et al. 2008 ), adult life span, survival, fecundity, and fertility ( Yang et al. 1994 ). As a result, temperature profoundly affects colonization, distribution, abundance, behavior, life history, and fitness of insects ( Hoffman et al. 2003 ). Therefore, information on the thermal requirements of intrusive insect pests’ development has important implications for control programs, as temperature determines the population growth and size of intrusive pests and their variation under different conditions ( Kang et al. 2009 ).

The melon thrips, *Thrips palmi* Karny (Thysanoptera: Thripidae), has become one of the most serious pests of eggplant, cucumber, field beans, melons, peppers, and flowers in Taiwan since its arrival in 1979 ( Chang 1995 ). It has been widely distributed around the world in subtropical and tropical regions ( Cannon et al. 2007 ). The life stages of *T. palmi* include the egg, two larval instars, two pupal instars, and the adult. The adult, egg, and the larval stages are found on the host plants. Pupae are found in the soil or among leaf litter. Females may lay up to 200 eggs in their two-month life span ( Zhang and Brown 2008 ). Both larvae and adults feed on leaves, flowers, and fruits, and cause scarring and deformities from extensive feeding. Fruits severely damaged by *T. palmi* have a low marketing value (5–10 NT$/kg) compared to good quality fruits (35–40 NT$/kg) in Taiwan. Capinera (2008) reported that the egg-to-adult developmental time of *T. palmi* is 16.5 days at 25° C and 42 days at 15° C.

Due to their complicated life cycle, *T. palmi* is difficult to control ( Cooper 1990 ; Kawai 1995; Lewis 1997 ; Kawai 2001 ; Cannon et al. 2007 ). Growers in Taiwan rely on various new insecticides to protect eggplant, *Solanum melongena* L. (Solanales: Solanaceae), from *T. palmi* , but most available insecticides provide unsatisfactory results. Since multiple applications of broad-spectrum insecticides eliminate natural enemies, target insects tend to develop resistance against frequently used insecticides. Forecasting models based on heat accumulation units should be developed for *T. palmi* control, as they could improve the timing of pesticide application and minimize overall use of pesticides. Therefore, the objective of this study was to find their development rate, and lower temperature threshold, survivorship, fecundity, longevity, sex ratio, and population trend index at different temperatures. Understanding the optimal temperatures for the major phenologicallyrelated parameters of *T. palmi* (e.g., developmental, fecundity, and longevity) would be helpful to the efficient control of *T. palmi* in Taiwan.

## Materials and Methods

### Laboratory rearing of *T. palmi*

**Rearing conditions.** Life table studies were conducted at 16, 19, 22, 25, 28, and 31° C with 70–80% RH and a 12:12 L:D photoperiod on eggplant leaves in National Pingtung University of Science and Technology, Taiwan.

**Insect rearing.** Leaves containing *T. palmi* males and females were collected from a farmer’s field in Pingtung County, southern Taiwan. By using a dissecting microscope (20×), pairs of males and females from infested leaves were transferred to petri dishes (9 cm diameter) containing healthy eggplant leaves (3×3 cm). Water soaked filter paper was placed at the bottom of each petri dish to prevent desiccation of leaflets. The petri dishes containing a male and female pair were kept in an incubator at 25° C for eight hours. In order to maintain sufficient egg laying stock, a total of 10 pairs were placed in the incubator for each temperature studied. The eggplant leaves were examined under a dissecting microscope (20×), and approximately 120, 160, 160, 160, 160, and 130 eggs, along with leaf sections, were placed in an incubator at 16, 19, 22, 25, 28, and 31° C, respectively. The egg development period was determined by appearance of larva; however, egg mortality could not be determined. The duration of egg development was noted at each temperature.

When first instar larvae appeared, a single larva was placed in each Petri dish (9 cm diameter) containing an eggplant leaf. The Petri dish was sealed with Parafilm and placed in the growth chamber. In this way 120 larvae were placed in an incubator at 16° C, 160 larvae at 19° C, 160 larvae at 22° C, 160 larvae at 25° C, 160 larvae at 28° C, and 130 larvae at 31° C. Moist cotton was placed in each petri dish for maintaining sufficient moisture. Leaves were transferred every other day. Observations were taken every 24 hours. The duration of developmental time at each stage was recorded separately for each temperature. The stages considered were egg, first and second instar larvae, total pupal stage (prepupae + pupae), and adult.

### Effect of temperature on fecundity, oviposition, and longevity

After emergence of adults, each *T. palmi* pair was placed separately on eggplant leaves (3 × 3 cm) in a petri dish (9 cm diameter) containing moist filter paper. The petri dishes containing male and female *Thrips palmi* were placed in separate growth chambers for egg laying. The leaves with eggs laid on them were replaced daily with new leaves until the female adult died. A total of 39, 50, 68, 78, 60, and 59 new-born females were studied for fecundity at 16, 19, 22, 25, 28, and 31° C, respectively. Fecundity and survival were recorded daily until the death of each female at every temperature. The longevity of male and female adults was measured in all temperature condition.

### Temperature threshold and thermal constant for preimaginal development

The relationship between temperature and development rate (the reciprocal of developmental time in days) was determined by the linear regression model *V* = *a* + *bT* , where *V* development rate, *T* is temperature, and *a* and *b* are constants that were estimated by least square regression analysis using SPSS version 18 ( SPSS 2010 ). The linear model allows the determination of the lower threshold temperature and the thermal constant, i.e., the amounts of thermal units (degreedays) required to complete preimaginal development. The accumulated effective temperature (Kelvin ( *K* )) was estimated as *K* = 1/ *b* , and lower temperature threshold was estimated as – *a/b* ( Campbell et al. 1974 ).

### Data analysis

Differences in developmental time, fecundity, longevity, adult preoviposition period, and total preoviposition period among temperature treatments were tested by oneway analysis of variance (ANOVA). If significant differences were detected, multiple comparisons were performed using Waller-Duncan’s range tests and Scheffe’s test. Differences in longevity between females and males were analyzed by ANOVA and Student’s *t* -test (PASW Statistics 18.0.3 2010). The life table was constructed according to the Morris-Watt model ( Morris 1963 ), which is described as: *I* = *N1 / N0* = *SESSSLSPSAFPFP♀* , where *I* is the population trend index; *N1* and *N0* are the numbers of next generation and current generation, respectively; *SE* , *SS* , *SL* , *SP* are the respective survival rates of eggs, first instar larvae, second instar larvae, and pupae; *SA* is the survival rate of adults; *F* is the number of initial eggs; *PF* is the average number of eggs laid per female; and *P♀* is the proportion of adults that are female.

## Results

**Figure 1.**
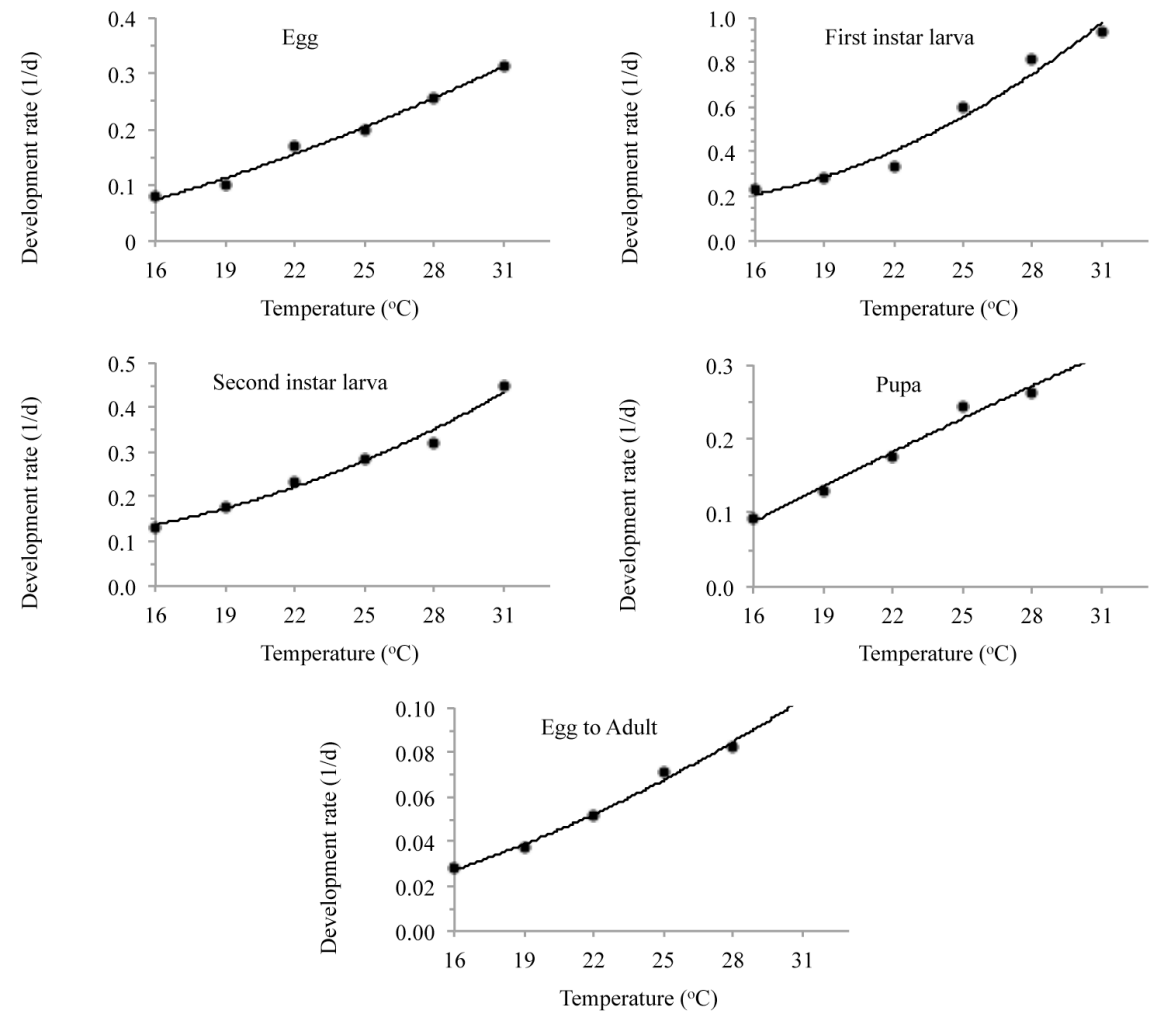
Developmental rate (1/ *d* ) of *Thrips palmi* at different constant temperatures (curves are described by a polynomial model). High quality figures are available online.

The developmental times for each stage of *T. palmi* at six temperatures are presented in Table 1 . The developmental time for each stage was significantly shortened as the temperature increased (eggs: *F* 5, 864 = 5679.65, df = 5, 864, *p* < 0.0001; first instar larvae: *F* 5,826 = 1136.08, df = 5, 826, *p* < 0.0001; second instar larvae: *F* 5,724 = 1259.57, df = 5, 724, *p* < 0.0001; pupae: *F* 5,583 = 1726.95, df = 5, 583, *p* < 0.0001; preadult: *F* 5,583 = 6237.43, df = 5, 583, *p* < 0.0001). The mean developmental time of eggs decreased with the temperature increase from 16° C to 31° C ( Table 1 ). Consequently, the overall duration of development from egg to adult was longest at 16° C and shortest at 31° C (egg to adult development: *F* 5, 583 = 6237.43, df = 5, 583, *p* < 0.0001) ( Table 1 ). The data on developmental time and the relationship between temperature and developmental rate are described by a polynomial model ( Figure 1 ).

**Table 1.**
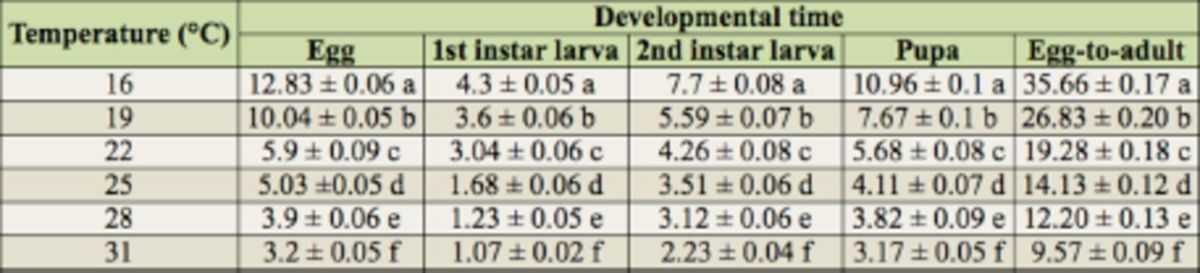
Developmental time for each stage of *Thrips palmi* at six constant temperatures.

† Means within the same column followed by a different letter are significantly different at *p* < 0.0001 (ANOVA followed by Duncan’s new multiple range test).

**Figure 2.**
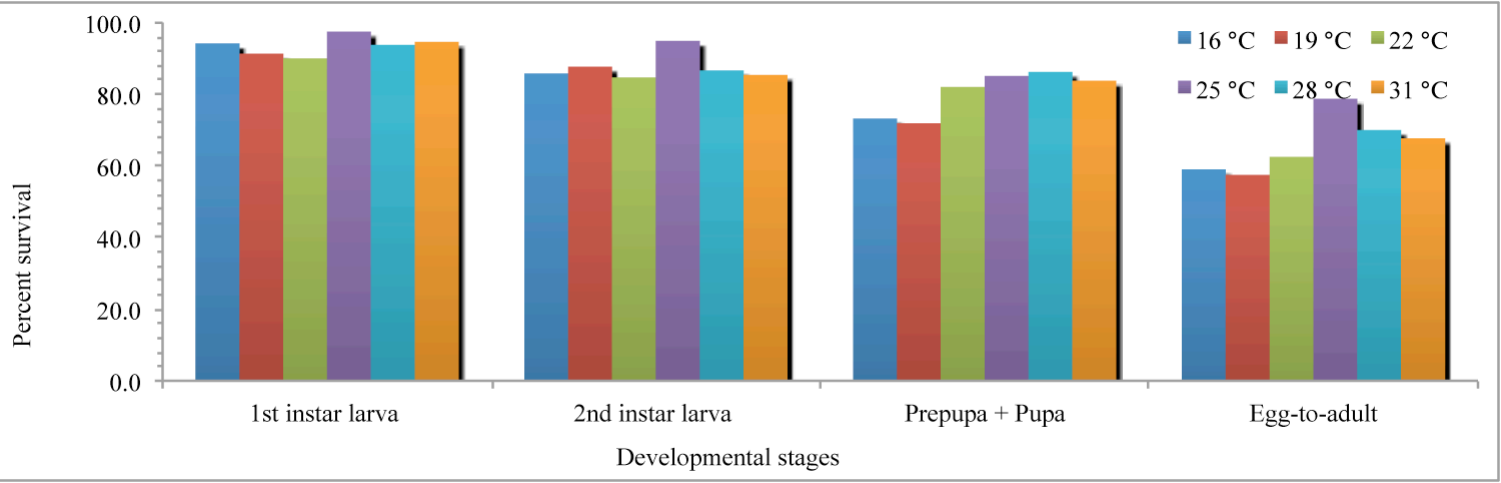
Survival of various stages of *Thrips palmi* at six constant temperatures on eggplant. High quality figures are available online.

The maximum survival rate of first instar larvae (97.5%) and second instar larvae (94.9%) was observed at 25° C; however, pupal survival rate was highest (86.1%) at 28° C ( Figure 2 ). The survival rate of first instar larvae ranged from 90 to 97.5%, whereas that of second instar ranged from 84.7 to 94.9%. Pupal survival ranged from 71.9 to 86.2% at different temperatures. Within the temperature range from 16 to 31° C, emergence rate from egg-to-adult ranged from 57.5 to 78.8%, and differed significantly ( *t* = 20.3, df = 5, *p* < 0.0001). A significantly lower percentage of individual (57.5%) completed egg-to-adult development at 19° C, and the highest percentage (78.8%) occurred at 25° C ( Figure 2 )

**Table 2.**
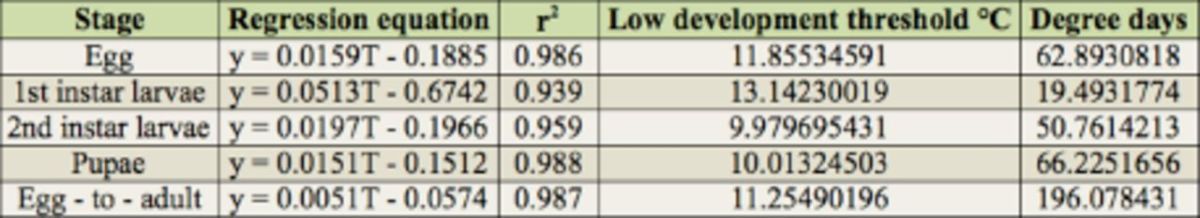
Low developmental threshold temperatures and thermal requirements for preimaginal development of *Thrips palmi*

### Temperature threshold and thermal requirements for preimaginal development

Estimates of the threshold temperature and the thermal constant based on the linear regression equation are presented in Tables 2 and 3 . The preimaginal developmental rates were linearly related to temperature for the entire range of temperatures tested, from 16 to 31° C. The theoretical threshold temperature for total preimaginal development was 11.25° C, whereas the thermal requirements for preimaginal development were 196.1 degreedays ( Table 2 ). The highest threshold temperature for development was noted for first instar larvae (13.14° C). Mean fecundity of *T. palmi* was significantly influenced by temperature ( *F* 5, 348 = 510.09, df = 5, 348, *p* < 0.0001), with the highest fecundity occurring at 25° C and the lowest at 16° C ( Table 4 ). Within the temperature range of 16 to 31° C, the relationship between the number of eggs laid and temperature followed a parabolic pattern: y = -0.3 *x2* + 16.4x – 168.2 ( *r2* = 0.83, *p* < 0.05); where y is the number of eggs laid, and x is the temperature. When first-order derivative of the equation equals zero, the value of x equals 27.33° C. Therefore, the maximum egg laying temperature was estimated to be 27.33° C.

**Table 3.**
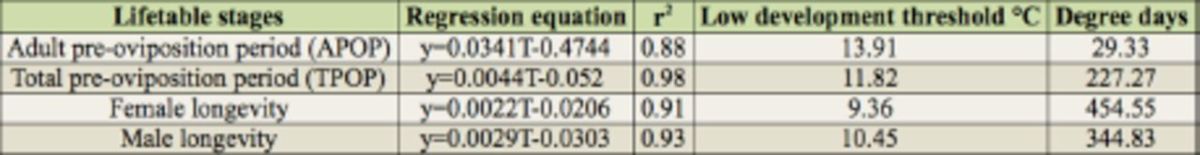
Low developmental threshold temperatures and thermal requirements for adult preoviposition period, total preoviposition period, and longevity of *Thrips palmi.*

**Table 4.**
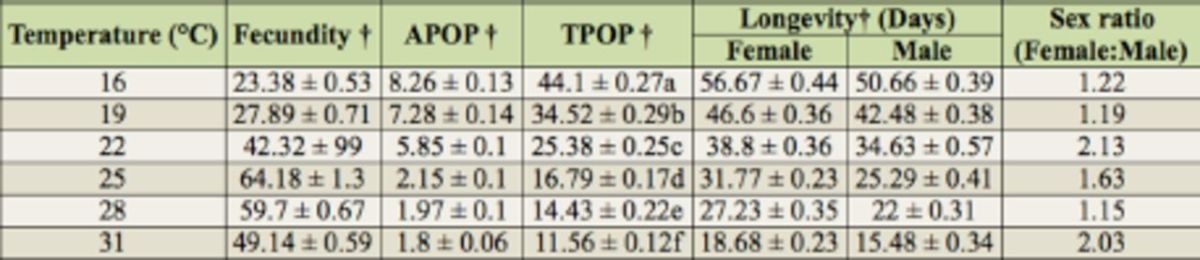
Fecundity, longevity, adult preoviposition period (APOP), total preoviposition period (TPOP), and sex ratio of *Thrips palmi* at six constant temperatures.

† Data show the mean ± SE. Means within the same column followed by different letters are significantly different at *p* < 0.0001 (ANOVA followed by Duncan’s new multiple range test).

### Effect of temperature on oviposition, longevity, and sex ratio

Female longevity was significantly higher than male longevity at all temperatures (16° C: *t* = 10.049, df = 69, *p* < 0.0001;19° C: *t* = 11.37, df = 90, *p* < 0.0001; 22° C : *t* = 9.07, df = 98, *p* < 0.0001; 25° C: *t* = 21.09, df = 124, *p* < 0.0001; 28° C: *t* = 15.65, df = 110, *p* < 0.0001; 31° C: *t* = 7.78, df = 86, *p* < 0.0001). Longevity of both females and males was significantly longest at 16° C (female: *F* 5, 348 = 2473.13, df = 5, 348, *p* < 0.0001; male: *F* 5,229 = 1717.84, df = 5, 229, *p* < 0.0001) and the shortest at 31° C ( Table 4 ). The adult preoviposition period and total preoviposition period of the female adults significantly decreased (adult: *F* 5, 348=1217.21, df = 5, 348, *p* < 0.0001; total: *F* 5, 348 = 4736.9, df = 5, 348, *p* < 0.0001) with increasing temperature ( Table 4 ). The adult preoviposition periods at 25 and 28° C were not significantly different; however, they were significantly different at all other temperatures. The adult preoviposition period was longest at 16° C and shortest at 31° C. The estimated theoretical threshold temperature for the adult preoviposition period was 11.82° C, and the respective heat unit requirement was 227.27 degreedays ( Table 3 ).

The sex ratio of the progeny females that completed their preimaginal development at the different temperatures tested ranged from 1.15 to 2.13 and was significantly affected by rearing temperature ( *t* = 8.661; df = 5 *p* < 0.0001). The sex ratio (female/male) of adults was highest at 22° C and the lowest at 28° C.

### Life table and population trend index

Life tables of *T. palmi* were constructed based on survival rate, fecundity, and sex ratio ( Table 5 ). Because *T. palmi* eggs are laid inside the leaf tissue, the mortality of eggs was not determined. The hatching rate of eggs was considered to be 100%. The survival rate of first instar larvae, second instar larvae, and pupae, and female proportion were based on actual data, as described in Table 5 . Population trend index measures the potential of population growth between the next generation and the current generation. Temperature significantly affected the population trend index at different temperatures ( *t* = 4.86, df = 5, *p* = 0.005). The index value was highest at 25° C ( Table 5 ). The results showed that the population increased 31.34 times after one generation at 25° C, thus showing this temperature to be optimal for *T. palmi* population growth.

**Table 5.**
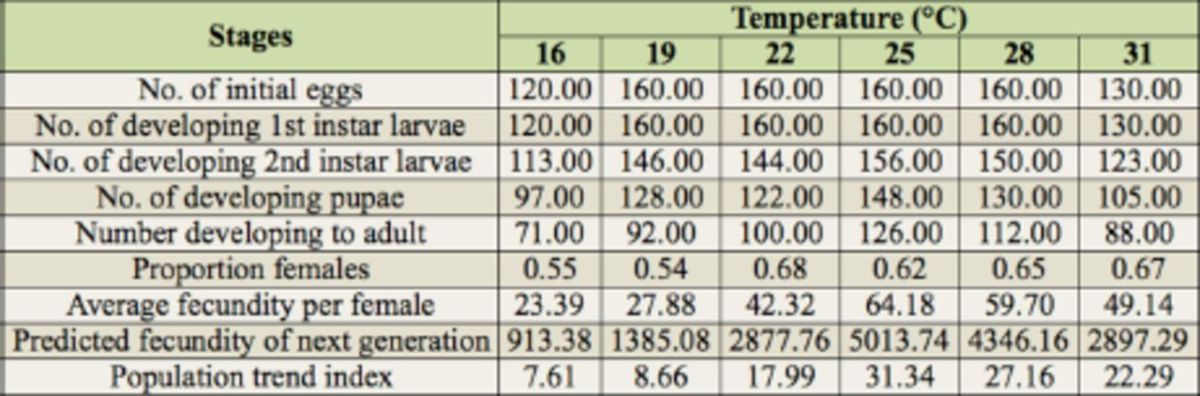
Life table of *Thrips palmi* at six constant temperatures.

## Discussion

Temperature is the most important abiotic factor affecting insect growth, development rate, and survival of *T. palmi* . Results from this study indicated that the mean developmental time of different stages of *T. palmi* declined with increasing temperature. Park et al. (2010) reported the egg-to-adult mean development times of *T. palmi* at different temperatures on cucumber were 40.8, 20.5, 12.7, and 9.8 days at 15, 20, 25, and 30° C, respectively. However, Tsai et al. (1995) documented preadult development times of 43.3, 16.4, and 10.9 days at 15, 26, and 32° C on eggplant. McDonald et al. (1999) reported the total preimaginal development periods for *T. palmi* were 40.2, 16.6, 15.2, and 10.1 days at 15, 21, 23, and 30° C in germinated bean seeds. However, our study of *T. palmi* in eggplant indicated egg-to-adult development periods of 35.66, 26.83, 19.28, 14.13, 12.20, and 9.57 days at 16, 19, 22, 25, 28, and 31° C. These relatively minor differences in the preimaginal development periods might be due to differences in host plants, rearing conditions, and observation intervals.

The lower threshold for development and the thermal constant are useful indicators for an insect’s potential distribution ( Campbell et al. 1974 ). Our findings showed that a threshold temperature of 11.25° C and 196.1 degreedays were required for *T. palmi* to complete one generation. From this, it is concluded that *T. palmi* could possess the ability to remain active in the short winters in Taiwan. The estimated threshold temperature and thermal constant for preimaginal development found in our study are nearly similar to Kawai (1990) . Kawai (1990) documented the estimated threshold temperature of 11.6° C and the thermal constant of 189.1 degreedays for total preimaginal development of *T. palmi* in cucumber. However, according to Park et al. (2010) , the developmental thresholds for egg, larvae, prepupae, and pupae were10.6, 10.6, 9.1, and 10.7° C, respectively, in cucumber plants. In our study, developmental thresholds for egg, first instar larvae, second instar larvae, and pupae were 11.81, 13.14, 9.90, and 10.07° C, respectively, which is slightly different than Park et al. (2010) .

Temperature had a significant effect on egg production of *T. palmi* ( Table 4 ). In our study, the number of eggs per female reached their highest values at 25° C (64.18 eggs per female). This result indicates that 25° C is the optimal temperature for reproduction. Based on the fecundity per female at different temperatures, the optimum temperature for maximum egg-laying was estimated to be 27.33° C by polynomial regression. Capinera (2008) reported that female *T. palmi* can produce up to 200 eggs. However, Tsai et al. (1995) reported 29.0 eggs per female of *T. palmi* in eggplant. Zhang and Brown (2008) documented *T. palmi* can lay up to 100 eggs.

The current results indicate that longevity, adult pre-oviposion period, and total preoviposition periods were extended with decreasing temperature ( Table 4 ). Capinera (2008) reported that the adult longevity of females was longer (10–30 days) than males (7– 20 days). Tsai et al. (1995) documented the adult longevity of females and males of *T. palmi* significantly declined as the temperature increased from 15 to 32° C. According to Tsai et al., adult females had longer longevity (13–24 days) than males (11.1–13.7 days). The mean adult preoviposition period decreased from 8.26 days at 16° C to 1.8 days at 31° C, and the total preoviposition period decreased from 44.1 days at 16° C to 11.56 days at 31° C. This finding shows that *T. palmi* can quickly build-up its population at higher temperatures as compared to lower temperature. At all the tested temperatures between 16 and 31° C, the proportion of female emergence was significantly higher than male population emergence. The higher emergence of females than males indicates that *T. palmi* can easily build-up a large population in the range of the experimental temperatures.

The percentage of *T. palmi* completing preimaginal development was significantly affected by temperature. The percentage of *T. palmi* that completed egg-to-adult development varied from 57.5 to 78.75% ( Table 5 ). These findings suggest that *T. palmi* could survive in short, mild winters in Taiwan and can build-up high populations in the summer. The higher emergence rate from egg-to-adult (67.7 to 78.75%) in the range of 25 to 31° C might be the cause of the serious damage to eggplant in the summer season in Taiwan.

Bellows et al. (1992) explained that life tables are powerful tools to indicate growth, survival, reproduction, and growth rate of an insect population. In addition, the classical life table is primarily used to understand the age dynamics of adult populations studied under control laboratory conditions. Data from our study indicated that temperature had a strong effect on life table parameters by affecting the survival, longevity, and fecundity of *T. palmi* . The population trend index was lowest (7.61) at 16° C and highest (31.34) at 25° C ( Table 5 ), showing that 25° C is the optimal temperature for the growth and establishment of *T. palmi* populations in Taiwan.

In summary, findings of this study provide fundamental information about development, survival, fecundity, and population trend index of *T. palmi* for assisting in predicting population growth and integrated pest management. Chemical insecticides are the most common practice for the management of *T. palmi* in Taiwan. Timing for pesticides treatments can be improved if the experimentally determined thermal requirements are used to predict seasonal emergence of *T. palmi* . The coldest months are from January to March in Taiwan, with the lowest temperature to about 10° C, which is near to threshold temperature for *T. palmi* emergence. The prediction suggests that the spray of insecticide application against *T. palmi* should be performed at this time, which may effectively control *T. palmi* in eggplant.
